# Relationship between Signals Regulating Energy Homeostasis and Neuropsychological and Clinical Features in Gambling Disorder: A Case-Control Study

**DOI:** 10.1192/j.eurpsy.2023.722

**Published:** 2023-07-19

**Authors:** F. Fernandez-Aranda, I. Baenas, M. Etxandi, B. Mora-Maltas, R. Granero, S. Tovar, C. Diéguez, M. N. Potenza, S. Jiménez-Murcia

**Affiliations:** ^1^Psychiatry, Bellvitge University Hospital, Barcelona; ^2^Ciber Fisiopatología Obesidad y Nutrición, Madrid; ^3^ Psychoneurobiology of Eating and Addictive Behaviors Group, Bellvitge Institute for Biomedical Research; ^4^Clinical Sciences, Medicine and Health Sciences School, University of Barcelona, Barcelona; ^5^Psychiatry, Hospital Universitari Germans Trias i Pujol, IGTP Campus Can Ruti, Badalona; ^6^Psychobiology and Methodology, Autonomous University of Barcelona, Barcelona; ^7^Physiology, CIMUS, University of Santiago de Compostela, Instituto de Investigación Sanitaria, Santiago de Compostela, Spain; ^8^Psychiatry; ^9^Child Study Center, Yale University Medicine School; ^10^Connecticut Mental Health Center, New Haven, CT; ^11^Connecticut Council on Problem Gambling, Wethersfield, CT; ^12^Neuroscience, Yale University, New Haven, CT, United States

## Abstract

**Introduction:**

The neurobiology of gambling disorder (GD) is not yet fully understood. Although dysfunctional signalling involved in energy homeostasis has been studied in substance use disorders, it should be examined in detail in GD.

**Objectives:**

To compare different endocrine and neuropsychological factors between individuals with GD and healthy controls (HC), and to explore endocrine interactions with neuropsychological and clinical variables.

**Methods:**

A case-control design was performed in 297 individuals with GD and 41 HC, assessed through a semi-structured clinical interview and a psychometric battery, adding 38 HC in the evaluation of endocrine and anthropometric variables.

**Results:**

Individuals with GD presented higher fasting plasma ghrelin (p<.001) and lower LEAP2 and adiponectin concentrations (p<.001) than HC adjusting for body mass index (BMI). The GD group reported higher cognitive impairment regarding cognitive flexibility and decision-making strategies, a worse psychological state, higher impulsivity levels, and a more dysfunctional personality profile. Despite failing to find significant associations between endocrine factors and either neuropsychological or clinical aspects in GD, some impaired cognitive dimensions and lower LEAP2 concentrations significantly predicted GD presence.

**Conclusions:**

This study suggests distinctive neuropsychological and endocrine dysfunctions may operate in individuals with GD, predicting GD presence. Further exploration of endophenotypic vulnerability pathways in GD appear warranted, especially with respect to etiological and therapeutic potentials.

**Disclosure of Interest:**

F. Fernandez-Aranda Consultant of: Novo Nordisk , Employee of: editorial honoraria as EIC from Wiley, I. Baenas: None Declared, M. Etxandi : None Declared, B. Mora-Maltas: None Declared, R. Granero : None Declared, S. Tovar : None Declared, C. Diéguez: None Declared, M. Potenza Grant / Research support from: Mohegan Sun Casino and Connecticut Council on Problem Gambling, Consultant of: Opiant Pharmaceuticals, Idorsia Pharmaceuticals, Baria-Tek, AXA, Game Day Data and the Addiction Policy Forum; has participated in surveys, mailings or telephone consultations related to drug addiction, impulse control disorders or other health topics; and has consulted for law offices and gambling entities on issues related to impulse control or addictive disorders, Employee of: patent application in Yale University and Novartis, S. Jiménez-Murcia: None Declared.

